# Post-transcriptional control of gene expression by β-catenin: expanding the non-canonical ARMoury

**DOI:** 10.1038/s41388-025-03470-5

**Published:** 2025-06-25

**Authors:** O. Sevim, H. Park, R. G. Morgan

**Affiliations:** 1 School of Life Sciences, University of Sussex, Brighton, UK; 2 Brighton & Sussex Medical School, University of Sussex, Brighton, UK; 3 University Hospitals Sussex NHS Foundation Trust, Brighton, UK

**Keywords:** Cell biology, Physiology

## Abstract

The Wnt/β-catenin pathway is an evolutionarily conserved signal transduction cascade with critical regulatory roles in cellular proliferation, cell fate determination and tissue homeostasis. Through the regulation of multiple human stem cell systems, canonical Wnt signalling is not only a major contributor to normal development, but also heavily implicated in a multitude of human diseases, including cancer. The central mediator of the pathway β-catenin, first identified as Armadillo (ARM) in *Drosophila*, has well-defined roles in cell adhesion and transcription within the pathway. However, accumulating evidence suggests β-catenin functionality is more complex than initially anticipated with reported roles beyond those historically characterised, including the regulation of RNA and RNA-binding proteins (RBP). This review will summarise the current understanding around β-catenin as a post-transcriptional regulator in normal and malignant development, drawing particular attention to cell types not traditionally used to characterise Wnt signalling but uniquely placed to reveal novel β-catenin function.

## Introduction

### Canonical Wnt signalling

Canonical Wnt signalling activation is dependent on the engagement of extracellular Wnt ligands with the cognate Frizzled (FZD) and lipoprotein receptor-related protein (LRP5/6) co-receptors (recently superbly reviewed by Maurice & Angers) [1]. In their absence (termed ‘OFF-state’), β-catenin is constitutively targeted for cytoplasmic sequestration by a multiprotein destruction complex (DC) comprising two scaffold proteins, AXIN and adenomatous polyposis coli (APC), alongside two serine-threonine kinases, casein kinase 1α (CK1α) and glycogen synthase kinase 3β (GSK3β) [2]. β-Catenin is successively phosphorylated at Ser45 by CK1α and Thr41/Ser33/Ser37 in its phospho-degron within the N-terminus by GSK3β, promoting polyubiquitination via β-transducin repeat containing E3 ubiquitin protein (β-TrCP) and proteasomal degradation. Critically, this leads to the restriction of nuclear β-catenin and suppressed transcription of Wnt target genes, which remain bound by a repressive complex, including transducin-like enhancer (TLE) and Groucho. Together, these transcriptional co-repressors maintain the compaction of chromatin through the recruitment of histone deacetylases (HDACs) [3].

In contrast, engagement of FZD and LRP5/6 by Wnt ligands through autocrine/paracrine methods (termed ‘ON-state’) leads to the disassembly of the DC in a Dishevelled (DVL) dependent manner, leading to the cytoplasmic stabilisation of newly synthesised β-catenin. Following its translocation into the nucleus, β-catenin forms an essential component of the Wnt signalling enhanceosome, bringing together critical transcriptional co-activators such as p300 and CBP, through key adaptors including Pygo and BCL9, to activate the main T-cell Factor (TCF)/Lymphoid enhancer factor (LEF) family of Wnt transcriptional effectors. This leads to the activation of classic Wnt target genes such as *MYC* [4], *BIRC5* [5] and *CCND1* [6] which regulate critical biological processes including cell proliferation, differentiation, self-renewal and survival.

### β-Catenin structure

β-Catenin is the rate-limiting central mediator of the pathway, converting extracellular Wnt signals into downstream Wnt gene activation. The *CTNNB1* gene on chromosome 3p21 encodes a 781 amino-acid protein. It is a member of the broader catenin gene family composed of p120, beta and alpha, where strong structural and functional homology exists between the p120 subfamily (encompassing ARVCF, delta catenin, PKP1, PKP2, PKP3 and PKP4) and the beta subfamily (including β-catenin and γ-catenin) [7]. Structurally, β-catenin is composed of a central region of twelve highly basic repeating Armadillo (ARM) motifs which are flanked by an acidic N-terminal domain (NTD) and C-terminal domain (CTD). The NTD harbours a phospho-degron motif which serves as a site for phosphorylation, and various oncogenic mutations have been found to be enriched within this site leading to the abnormal stabilisation of β-catenin [8, 9]. The CTD contains a transactivation domain responsible for binding to several transcription and chromatin regulators, mediating the transcriptional function of β-catenin [10]. Each ARM repeat (~42 residues), termed R1-12, are formed from three α-helices which together serve as a rigid interaction platform for the vast majority of β-catenin’s binding partners at the membrane, cytosol and the nucleus which are comprehensively summarised on the Wnt homepage [11].

In certain instances, there exists mutual exclusivity in the capacity for β-catenin's partners to bind to the ARM domain, for example, E-cadherin, APC and TCF/LEF bind to a common region in R3-9 where salt bridges are formed with Lys312 and Lys435. Based on distinct charge distributions, it was proposed that the NTD and CTD may act to enhance the specificity of protein-protein interactions with the ARM domain [12]. Furthermore, directly distal to the 12^th^ ARM repeat lies a specific conserved helix (termed Helix-C), which is critical for the signalling activity of β-catenin, whilst being dispensable for its role in cell adhesion via adherens junctions as discussed below [12]. β-Catenin lacks canonical nuclear localisation or export sequences (NLS or NES) and therefore its cytoplasmic-nuclear trafficking is dependent on a variety of mechanisms including phosphorylation, molecular chaperones and compartmental retention which act in a tissue-dependent manner [13].

## Canonical β-catenin function

### Homotypic cell adhesion

**Fig. 1:**
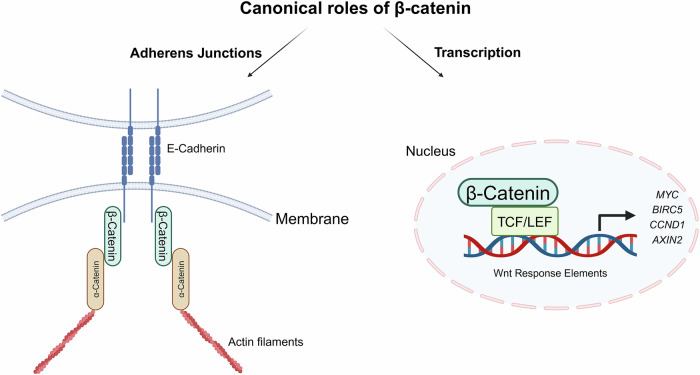
Schematic overview of well-established roles of β-catenin. β-Catenin plays a crucial role in adherens junctions by interacting with E-cadherin and α-catenin at the cell membrane, particularly in epithelial cells, to maintain structural integrity. When translocated to the nucleus following canonical Wnt signalling activation, β-catenin interacts with TCF/LEF transcription factors on Wnt-responsive elements to activate the transcription of Wnt target genes, functioning as a transcriptional co-activator.

β-Catenin has two very well-defined functions in cell adhesion and transcription (Fig. 1). In normal solid tissues, free β-catenin is immediately sequestered into adherens junctions (AJ) where it binds cadherins to the actin cytoskeleton (via additional catenins), to maintain homotypic cell adhesion, tissue polarity and cell integrity. Classical cadherins are single-pass transmembrane glycoproteins, which engage in Ca^2^^+^ mediated cell adhesion via their extracellular regions and bind to β-catenin through their cytoplasmic tails. Whilst β-catenin is known to interact with all forms of classical cadherins, the β-catenin-E-cadherin interaction remains the most extensively characterised. β-Catenin associates with E-cadherin in the endoplasmic reticulum and are subsequently transported to the cell membrane, which shields β-catenin from being targeted by the DC [14, 15]. In addition, the direct binding of α-catenin to β-catenin’s NTD enables the indirect modulation of the actin cytoskeleton [16], resulting in clustering of AJ proteins and eventually cell adhesion stabilisation [17]. The loss of the *CDH1* gene expression in solid tumours and subsequent release of signalling-competent β-catenin from AJs is one of the key initiating steps in promoting the epithelial-mesenchymal transition (EMT) necessary for metastatic progression [18].

### Transcriptional co-activation

Once translocated into the nucleus upon cytoplasmic stabilisation, β-catenin participates in the activation of Wnt transcriptional programmes. The lack of a DNA-binding domain necessitates the presence of DNA-binding partners for β-catenin’s localisation to target gene promoters [12]. In vertebrates, four paralogues, TCF7 (TCF1), TCF7L1 (TCF3), TCF7L2 (TCF4) and LEF-1 are transcription factors (TF) which partner with β-catenin and direct binding to specific DNA motifs (CCTTTGAT(G/C)) via their high mobility group domain [19]. In addition, some variants have a cysteine-rich domain known as C-clamp that can enhance the TCF/LEF association with the target sequences. However, it is worthy of note that β-catenin may exert transcriptional roles independently of TCF/LEF - recent data have revealed that β-catenin can interact with a variety of TFs, such as OCT4, TBX3, HIF1α, SMAD and SOX [20–23]. Utilising genomic analyses, Mukherjee et al. demonstrated the role of SOX TFs as recruiters of β-catenin to Wnt-responsive enhancers (WREs), where a Wnt-enhanceosome complex may initiate β-catenin/SOX dependent transcription in the absence of TCF/LEF [24].

## Post-transcriptional roles for β-catenin

**Fig. 2:**
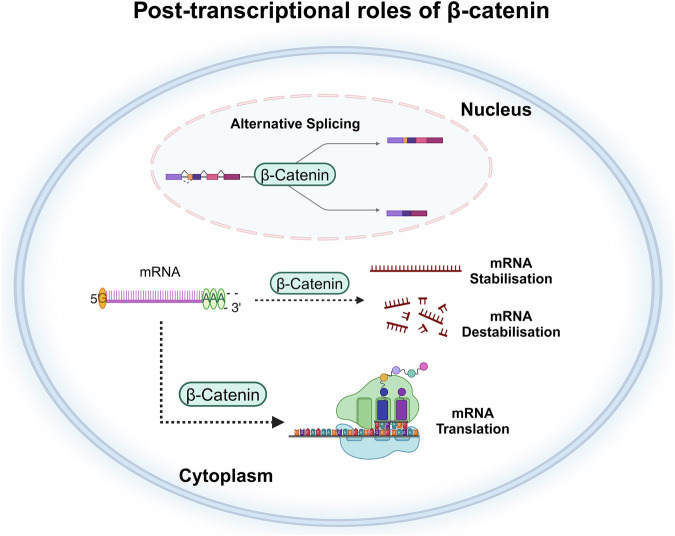
Schematic overview of β-catenin’s post-transcriptional roles. Data from the past two decades suggest that β-catenin may also serve as a post-transcriptional regulator in cells. Multiple reports have revealed novel roles of β-catenin in RNA metabolism, such as alternative splicing, mRNA stabilisation/destabilisation and translation. Regulatory functions of β-catenin in these processes give rise to the production of different isoforms through splicing, a change in mRNA half-lives through stabilisation or destabilisation of mRNAs and a change in protein production through mRNA translation.

In addition to its well-established roles in transcription and cell adhesion, many non-canonical roles have emerged for β-catenin including hormone internalisation, synaptic vesicle localisation, centrosome separation, SUMOylation, and exosome generation [25–28]. As previously shown for the TF WT1 [29] and more recently demonstrated for MYCN [30], emerging evidence also suggests a complex role for β-catenin gene expression through both transcriptional and post-transcriptional processes. Distinguishing between β-catenin’s well-established transcriptional role and any additional post-transcriptional influence will be challenging, and the remainder of this review is dedicated to the significant body of evidence highlighting a direct role for β-catenin in the post-transcriptional regulation of gene expression (Table 1 and Fig. 2).

**Table 1:** An overview of post-transcriptional roles mediated by β-catenin in various cell types.

mRNA targets	Function	Cell type	Reference
Adenovirus EA1 splicing reporter minigene	Alternative splicing	Fibroblast-like cells, colon cancer cells	Lee et al. (2007) [38] Sato et al. (2005) [31]
Oestrogen Receptor-β	Alternative splicing	Colon cancer cells	Lee et al. (2006) [32]
DGCR5	Alternative splicing	Oesophageal squamous cell carcinoma cells	Li et al. (2023) [33]
Cadherin 11	Stabilisation	Prostate cancer cells	Farina et al. (2009) [43]
CA9	Stabilisation	Breast cancer cells	D’Uva et al. (2013) [37]
SNAI2	Stabilisation	Breast cancer cells	D’Uva et al. (2013) [37]
TNF-α	Stabilisation	Breast cancer cells	Storci et al. (2013) [62]
IL-8	Stabilisation	Breast cancer cells	Storci et al. (2013) [62]
Pitx2	Stabilisation	Pituitary αT3-1 cells	Briata et al. (2003) [34]
Cyclin D1	Stabilisation	Colon cancer cells, pituitary αT3-1 cells	Briata et al. (2003) [34], Kim et al. (2012) [36], Lee et al. (2006) [32]
Cyclin D2	Stabilisation	Pituitary αT3-1 cells	Briata et al. (2003) [34]
c-Jun	Stabilisation	Pituitary αT3-1 cells	Briata et al. (2003) [34]
Cox-2	Stabilisation	Colon cancer cells	Kim et al. (2012) [36], Lee et al. (2006) [32]
VEGF-D	Destabilisation	Mouse fibroblasts	Orlandini et al. (2003) [42]
CA9	Transportation	Breast cancer cells	D’Uva et al. (2013) [37]

### Pre-mRNA splicing

The first reports that β-catenin may participate in post-transcriptional processes came with its association with splicing machinery in colorectal cancer cells. The identification of several splicing regulatory RNA-binding proteins (RBP) (e.g. FUS and TLS) in the nuclear β-catenin complex in colorectal adenocarcinoma cells (DLD-1), gave rise to the possibility that β-catenin may be involved in mRNA splicing [31]. To elucidate the impact of β-catenin on mRNA splicing, Sato et al. utilised the adenovirus *E1A* splicing reporter minigene, which has the potential to generate three major (13S, 12S, 9S) and two minor (11S and 10S) isoforms dependent on 5′-splice site selection. The authors reported that β-catenin transfection was able to modulate the splice site selection and promote the 10S isoform formation in Cos-7 cells in a dose-dependent manner [31]. In addition, β-catenin transfection in HeLa cells induced the expression of a novel 638-bp oestrogen receptor-β (ER-β) variant lacking exons 5 and 6 (ER-β Δ5-6), exerting dominant-negative activity, as well as splice variant of kallikrein 3 mRNA, which encodes protein-specific antigen (PSA) [31, 32].

Importantly, RNA co-immunoprecipitation coupled to RT-PCR demonstrated that β-catenin can directly bind to the ER-β transcript. The authors went on to utilise the systemic evolution of ligands by exponential enrichment (SELEX) protocol to generate a nuclear RNA aptamer against ARM 1–12 of β-catenin, which was able to repress the transcription of β-catenin target genes and a splicing variant driven by ectopic expression of β-catenin [32]. Finally, a recent report revealed that canonical Wnt signalling also promoted lncRNA splicing in oesophageal squamous cell carcinoma cells. Active nuclear β-catenin served as a co-factor for FUS to induce the spliceosome assembly on lncRNA-DGCR5 following Wnt3a stimulation, resulting in the generation of short isoform of lncRNA-DGCR5 rather than its long isoform [33].

### mRNA stability

In addition to splicing, β-catenin has also been implicated in regulating the stability of target/partner mRNAs. Activation of β-catenin was demonstrated to govern the stability of the mRNA of *Pitx2*, a transcriptional target of LEF-1, through modulating the subcellular localisation and interactive capacity of ARE-binding proteins (BPs) with AU-rich elements (AREs) in the 3′ untranslated region (UTR) in pituitary cells. ARE-BPs can either recruit or exclude exosomes to target transcripts and therefore are important mediators of RNA stability. Following Wnt induction, there was an increase in the cytoplasmic level and mRNA interaction of stabilising ARE-BP human antigen R (HuR), coupled with a decrease in the level of destabilising ARE-BPs, KSRP and TTP; furthermore, Pitx2 itself was shown to be capable of modulating the stability of additional Wnt-induced labile mRNAs, including *c-Jun, CyclinD1* and *CyclinD2*, at least in part mediated via HuR [34]. In keeping with this, β-catenin was shown to directly interact with the proximal region of the ARE of 3′-UTR of COX-2 mRNA both in vitro and in vivo in colorectal cancer cells, and induces its stabilisation co-ordinately via cytoplasmic localisation of HuR [35]. Mechanistically, Kim et al. identified an RNA binding motif (ACUUU) in the 3’ UTR of COX-2 mRNA which is recognised by the ARM domain of β-catenin; HuR binds to a non-overlapping sequence and together they form a tertiary RNP complex [36].

Furthermore, D’Uva et al. showed that β-catenin plays a pivotal role in the post-transcriptional machinery implicated in the hypoxia-induced de-differentiation programme in breast cancer. The authors demonstrated that β-catenin's accumulation in the cytosolic compartment in response to hypoxia exposure was critical in maintaining the stability of SNAI2 and CA9, two important stem cell regulatory genes. This was found to be in concert with HuR via interaction with 3′-UTR sequences, demonstrating that β-catenin’s ability to regulate post-transcriptional mechanisms exist in multiple tissue types [37]. In basal-like/triple negative breast cancer cells, β-catenin maintains the stability of epidermal growth factor receptor (EGFR)-governed genes, including interleukin-6 (IL-6). The paper also suggested that CD44, the hallmark of breast cancer stem cells, is also post-transcriptionally regulated by β-catenin, however, interestingly only HuR was found to bind to CD44 mRNA. In addition, Lee et al. also demonstrated that β-catenin can bind to COX-2 and CyclinD1 mRNA and increases their stability, suggestive of the possibility that β-catenin plays a role in multiple steps of gene expression [38].

In contrast, β-catenin has also been shown to inversely regulate vascular endothelial growth factor-D (VEGF-D) mRNA stability. VEGF-D is a secreted glycoprotein implicated in remodelling blood/lymphatic vasculature and has been associated with tumour progression in several human cancers [39–41]. Endogenous VEGF-D mRNA levels were down-regulated with constitutive expression of Wnt1 (Wnt/β-catenin signalling pathway agonist) and transfection of stable mutant β-catenin in mouse fibroblasts. On the other hand, siRNA mediated β-catenin depletion significantly induced VEGF-D mRNA levels. Through deletion analysis, the authors mapped the β-catenin-responsive mRNA destabilising element in the 3′-UTR to a conserved sequence corresponding to ARE group 1 [42]. Another destabilising effect of β-catenin was observed in the PC-3 prostate cancer cell line, which has endogenously highly active β-catenin expression levels. To determine β-catenin’s impact on cadherin-11 mRNA and protein levels, cadherin-11 3′ UTR reporters were transfected into PC-3 cells followed by β-catenin knockdown via siRNA. Removal of β-catenin led to significant induction in the activity and stability of cadherin-11 3′ UTR reporters. In addition, cadherin-11 steady state mRNA and protein levels was significantly induced in response to β-catenin knockdown [43].

Together, these data point towards several genes harbouring U/AU rich motifs at 3′-UTR to be under β-catenin dependent post-transcriptional control. This relationship across several tissue contexts and may act in concert with canonical RBP regulatory partners such as HuR which we also found in our β-catenin interactome studies in myeloid cells [44]. Further investigation into additional shared RNA-mediated interactions between β-catenin and HuR will aid the discovery of specific co-regulated transcripts.

### mRNA translation

β-Catenin interactome studies in alternative cell types beyond solid tumours harbouring Wnt signalling mutations have aided the discovery of further post-transcriptional roles. The β-catenin interaction network of vascular smooth muscle cells (VSMCs) identified 131 novel putative interacting partners, with Gene Ontology (GO) enrichment analyses revealing enrichment of mRNA translation/processing terms [45]. Following characterisation of a direct interaction between β-catenin and the fragile X mental retardation protein (FMRP), β-catenin was observed to join the pre-initiation complex in a FMRP-dependent fashion. Translation was also found to be repressed by β-catenin independent of its transcriptional activity. β-Catenin localisation from the cytoplasm to the nucleus in response to Wnt3a treatment gave rise to a significant increase in global translation in primary VSMCs [45]. This report suggests that β-catenin could suppress global translation under basal conditions in the cytoplasm, while the nuclear translocation of activated β-catenin away from the translational complex abrogates translational repression. More recently, the Bramham group demonstrated that phosphorylation of the eukaryotic initiation factor 4E (eIF4E) on Ser209 caused the release of translational repressors, and the recruitment of β-catenin, to the eIF4E cap complex. This led to the enhanced translation of mRNAs involved in critical cellular processes such as Notch signalling, MAPK signalling, ion transport and cell adhesion in dentate gyrus tissue [46]. In addition, β-catenin was reported to facilitate the transportation of CA9 and IL-6 mRNAs to the 40S ribosomal subunit to promote their translation in breast cancer cells [37].

### miRNA

β-Catenin also influences miRNA regulation. The *let-7* miRNA family are well-known tumour suppressors [47]. Interestingly, β-catenin was shown to affect *let-7* miRNA family expression in breast cancer stem cells. Stable β-catenin overexpression led to a significant reduction in the expression of mature *let-7* miRNA members, whilst the expression of mature *let-7* members was induced in response to removal of β-catenin. Notably, the changes in β-catenin protein in breast cancer stem cells did not affect the expression of primary *let-7* miRNAs, suggesting the impact of β-catenin on *let-7* miRNAs could be mediated through post-transcriptional mechanisms [48]. We recently identified the critical *let-7* regulator LIN28B as another RBP interaction partner for β-catenin in haematopoietic cells [44] (manuscript in preparation) raising the intriguing question of whether this represents a co-operative post-transcriptional axis governing miRNA expression. However, it is currently unknown whether β-catenin has the capacity to directly bind miRNAs and/or influence LIN28B:miRNA interactions.

### Focussed regulation of Wnt mRNAs

The haematopoietic system provides a unique environment with which to study non-canonical β-catenin function. Firstly, the well documented mutations to Wnt signalling regulators such as AXIN, APC, β-catenin or RNF43 are seldom reported in haematological malignancies. Furthermore, although β-catenin is well documented to serve transcriptional roles within this context, tight homotypic cell-cell adhesion is not a physiological feature of a fluid tissue, such as blood. Thus, proportionally less β-catenin is sequestered into cell adhesion complexes, increasing the pool of free β-catenin available to participate in alternative functions. Indeed, our seminal characterisation of the β-catenin interactome in haematopoietic cells revealed a plethora of novel protein partners including the significant enrichment of RBPs. These RBP partners were involved in multiple steps of RNA biogenesis including stability, splicing, and translation [44, 49, 50]. We have since validated a number of these including WT1 [49], MSI2 and HuR [50] with many others being further studied within our group. In addition, we have shown that β-catenin can affect the binding activity of MSI2 [50]. Interestingly, a similar approach to identifying β-catenin protein partners has recently been performed in T-ALL cells but using a different β-catenin antibody for co-immunoprecipitation. They also observed the significant enrichment of RBPs associated with ‘Spliceosome’, ‘RNA-binding’, ‘mRNA processing’, ‘mRNA transport’, ‘rRNA processing’ and ‘ribosome biogenesis’ through liquid chromatography-mass spectrometry analysis [51]. Many of the RBP partners identified in our study had clustered function around ‘*mRNA processing and transport*’ prompting us to explore the mRNA network associated with β-catenin.

Through β-catenin RNA-immunoprecipitation coupled to RNA sequencing (RIPseq) analyses in myeloid cell lines, we revealed a network of completely novel mRNAs interacting with β-catenin which included the enrichment of transcripts encoding Wnt signalling transcripts e.g. *AXIN2*, *LEF1*, *BCL9L*, *AMER1*, *TCF7* and *CSNK1E* [52]. We further went on to demonstrate that β-catenin likely binds one of these mRNAs, *LEF1*, in concert with the well-established RBP MSI2 and which could further impact the half-life of the *LEF1* transcript, and such an axis could regulate the growth/survival of human CD34^+^ haematopoietic stem/progenitor cells (HSPC).

Our study was the first to demonstrate β-catenin's interaction with RBPs and Wnt signalling mRNAs in a haematological context, however, the concept of post-transcriptional regulation of Wnt signalling components by β-catenin had already been established through the Bramham study mentioned above. Within the murine dentate gyrus, another cellular context free of stabilising Wnt mutations, Patil and co-workers demonstrated that β-catenin recruitment to the eIF4E cap complex led to the specific and preferential translation of Wnt signalling mRNAs including *Wnt4*, *Lrp5*, *Fzd2*, *Fzd4* and *Dvl2*. Collectively, these studies raise the fascinating prospect that in addition to its well-established role governing transcriptional feedback loops within Wnt signalling, β-catenin may well also regulate critical Wnt signalling feedback loops via post-transcription mechanisms.

### RNA binding

The involvement of β-catenin in post-transcriptional mechanisms begs the question of whether β-catenin is able to bind to RNA directly, or indirectly via its numerous RBP intermediates. The evidence for direct β-catenin:RNA interactions remain a little unclear currently.

The first evidence that β-catenin might bind RNA directly came from Lee et al. showing an RNA aptamer association with β-catenin. RNA aptamers are RNA oligonucleotides that can bind to a specific molecule with high affinity and specificity. They identified an RNA aptamer sequence binding to β-catenin, and RNA-Electrophoretic Mobility Shift Assay (EMSA) was also performed to display this RNA aptamer binding to β-catenin with the binding affinity (*K*_d_) of 5 nmol/L. In addition, this specific RNA aptamer was shown to bind to a particular region of ARM repeats, but not to bind to RNA binding protein HuR using RNA-EMSA, indicating the sequence specificity for the β-catenin molecule [32]. The same group subsequently showed that the ARM repeats of β-catenin recognised a specific RNA motif (ACUUU) for RNA aptamer binding. Furthermore, they revealed the existence of a similar motif in 3′-UTR region of COX-2 mRNA and showed that β-catenin was able to associate directly with 3′-UTR of COX-2 mRNA through recognition of the ACUUU motif [36].

RBPs are the critical post-transcriptional effectors of gene expression through modulating the temporal, spatial and functional dynamics of RNAs. In excess of 1500 RBPs have been annotated to date and have been characterised to modulate interacting RNA fate via a variety of mechanisms, including RNA splicing, polyadenylation, subcellular localisation, stability and translation. Canonical RBPs are defined by the presence of repetitive sequences, termed RNA binding domains (RBDs), which have the capacity to bind to specific structural RNA motifs. The best characterised of the more than 40 RBDs that have been described to date include RNA-recognition motif (RRM), hnRNP-K homology (KH) domain, double stranded RBD (dsRBD) and zinc finger domains; specificity for their RNA target is achieved via the synergistic effects of multiple domains [53]. β-Catenin lacks any canonical RNA binding motif and would thus be termed a non-canonical RBP, which lack traditionally defined RBDs but crucially retain the capacity for protein-RNA complex formation [10, 54]. As described above, β-catenin directly interacts with the AU-rich elements of 3′-UTRs and can influence various aspects of RNA metabolism, including alternative splicing of pre-mRNAs, mRNA stability, miRNA regulation, as well as translation. Furthermore, there is significant homology between ARM repeats of β-catenin and the PUF protein family (composed of Pumilio and FBF), which are established RBPs highly conserved in eukaryotes [55]. Moore et al. carried out a retrospective analysis to identify unconventional RNA binding domains associated with RNA binding, and ARM repeats were identified as the most common unconventional RNA binding domain that can bind to RNA amongst non-canonical RBPs [56], supporting the notion that β-catenin might be able to interact directly with RNA molecules through the 12 ARM repeats housed within its central domain. Recent findings have also demonstrated that ARM motifs are capable of binding double-stranded RNA [57].

To complicate the picture further, intrinsically disordered regions (IDR) of proteins also have high potential for direct RNA binding amongst non-canonical RBPs [58]. The N and C-termini of β-catenin contain IDRs and thus could serve as sites for RNA binding. In our most recent studies in haematopoietic cells, we were consistently able to isolate substantial concentrations of RNA from β-catenin RIP assays, however we were unable to precipitate any discernible RNA above background level using β-catenin cross-linking immunoprecipitation (CLIP), a more stringent evaluation of direct RNA-protein complexes [59]. This data would imply β-catenin’s interaction with RNA might be more indirect via its many RBP partners e.g. MSI2 and FMRP [45, 50].

## Conclusion

Efforts to drug β-catenin in cancer have largely focused on disrupting its interactions with key transcriptional partners such as TCF/LEFs or CBP/p300 but not yielded the anticipated efficacy. However, mounting evidence suggests β-catenin’s influence over gene expression is more holistic than previously understood and extends into the post-transcriptional regulatory landscape. Previous reports of β-catenin interaction with RBPs, RNA and numerous post-transcriptional processes including mRNA splicing, stability, translation and miRNA regulation indicate that it may have proved overly simplistic to simply target β-catenin’s transcriptional activity. The use of cell types not traditionally used for β-catenin characterisation that are free of Wnt mutations and/or competing cell-cell adhesion complexes, coupled with ever improving RNA based methodology, have reshaped our understanding of β-catenin activity and revealed novel non-canonical function.

A key challenge for the field now is the ability to precisely discriminate β-catenin’s transcriptional activity from its post-transcriptional influence, especially in light of recent data from leukaemia cells showing β-catenin can also direct an RNA processing and biogenesis programme through its transcriptional activity [60]. Furthermore, recent evidence suggests its transcriptional and post-transcriptional activities could be very tightly coupled given the observation of both nascent RNA and active RNA polymerase II within β-catenin condensates [61]. It would also be important to determine if any of β-catenin well-defined post-translational modifications moderate its post-transcriptional activity, independent of impacting its stability. Complete elucidation of β-catenin’s DNA and RNA-regulating activities will facilitate the design of novel and highly targeted next generation therapies that will specifically and efficiently eradicate oncogenic β-catenin activity.

## References

[1] Maurice MM, Angers S. Mechanistic insights into Wnt-β-catenin pathway activation and signal transduction. Nat Rev Mol Cell Biol. 2025;26:371–88. 39856369 10.1038/s41580-024-00823-y

[2] Ackers I, Malgor R. Interrelationship of canonical and non-canonical Wnt signalling pathways in chronic metabolic diseases. Diab Vasc Dis Res. 2018;15:3–13. 29113510 10.1177/1479164117738442 PMC5752873

[3] Sekiya T, Zaret KS. Repression by Groucho/TLE/Grg proteins: genomic site recruitment generates compacted chromatin in vitro and impairs activator binding in vivo. Mol Cell. 2007;28:291–303. 17964267 10.1016/j.molcel.2007.10.002 PMC2083644

[4] He TC, Sparks AB, Rago C, Hermeking H, Zawel L, da Costa LT, et al. Identification of c-MYC as a target of the APC pathway. Science. 1998;281:1509–12. 9727977 10.1126/science.281.5382.1509

[5] Zhang T, Otevrel T, Gao Z, Gao Z, Ehrlich SM, Fields JZ, et al. Evidence that APC regulates survivin expression: a possible mechanism contributing to the stem cell origin of colon cancer. Cancer Res. 2001;61:8664–7. 11751382

[6] Shtutman M, Zhurinsky J, Simcha I, Albanese C, D’Amico M, Pestell R, et al. The cyclin D1 gene is a target of the beta-catenin/LEF-1 pathway. Proc Natl Acad Sci USA. 1999;96:5522–7. 10318916 10.1073/pnas.96.10.5522 PMC21892

[7] Zhao ZM, Reynolds AB, Gaucher EA. The evolutionary history of the catenin gene family during metazoan evolution. BMC Evol Biol. 2011;11:198. 21740572 10.1186/1471-2148-11-198 PMC3141441

[8] Yumimoto K, Yamauchi Y, Nakayama KI. F-box proteins and cancer. Cancers. 2020;12:1249. 10.3390/cancers12051249 PMC7281081 32429232

[9] Uyar B, Weatheritt RJ, Dinkel H, Davey NE, Gibson TJ. Proteome-wide analysis of human disease mutations in short linear motifs: neglected players in cancer? Mol Biosyst. 2014;10:2626–42. 25057855 10.1039/c4mb00290c PMC4306509

[10] Hur J, Jeong S. Multitasking β-catenin: from adhesion and transcription to RNA regulation. Anim Cells Syst. 2013;17:299–305.

[11] Nusse R. The Wnt Homepage. 2023. https://web.stanford.edu/group/nusselab/cgi-bin/wnt/protein_interactions.

[12] Xing Y, Takemaru K, Liu J, Berndt JD, Zheng JJ, Moon RT, et al. Crystal structure of a full-length beta-catenin. Structure. 2008;16:478–87. 18334222 10.1016/j.str.2007.12.021 PMC4267759

[13] Morgan RG, Ridsdale J, Tonks A, Darley RL. Factors affecting the nuclear localization of β-catenin in normal and malignant tissue. J Cell Biochem. 2014;115:1351–61. 24610469 10.1002/jcb.24803

[14] Valenta T, Hausmann G, Basler K. The many faces and functions of β‐catenin. EMBO J. 2012;31:2714–36. 22617422 10.1038/emboj.2012.150 PMC3380220

[15] Huber AH, Weis WI. The Structure of the β-Catenin/E-Cadherin Complex and the Molecular Basis of Diverse Ligand Recognition by β-Catenin. Cell. 2001;105:391–402. 11348595 10.1016/s0092-8674(01)00330-0

[16] Nelson WJ. Regulation of cell–cell adhesion by the cadherin–catenin complex. Biochem Soc Trans. 2008;36:149–55. 18363555 10.1042/BST0360149 PMC3368607

[17] Wang B, Li X, Liu L, Wang M. β-Catenin: oncogenic role and therapeutic target in cervical cancer. Biol Res. 2020;53:33. 32758292 10.1186/s40659-020-00301-7 PMC7405349

[18] Thiery JP, Acloque H, Huang RY, Nieto MA. Epithelial-mesenchymal transitions in development and disease. Cell. 2009;139:871–90. 19945376 10.1016/j.cell.2009.11.007

[19] van Beest M, Dooijes D, van De Wetering M, Kjaerulff S, Bonvin A, Nielsen O, et al. Sequence-specific high mobility group box factors recognize 10-12-base pair minor groove motifs. J Biol Chem. 2000;275:27266–73. 10867006 10.1074/jbc.M004102200

[20] Kaidi A, Williams AC, Paraskeva C. Interaction between beta-catenin and HIF-1 promotes cellular adaptation to hypoxia. Nat Cell Biol. 2007;9:210–7. 17220880 10.1038/ncb1534

[21] Funa NS, Schachter KA, Lerdrup M, Ekberg J, Hess K, Dietrich N, et al. β-Catenin Regulates Primitive Streak Induction through Collaborative Interactions with SMAD2/SMAD3 and OCT4. Cell Stem Cell. 2015;16:639–52. 25921273 10.1016/j.stem.2015.03.008

[22] Kelly KF, Ng DY, Jayakumaran G, Wood GA, Koide H, Doble BW. β-catenin enhances Oct-4 activity and reinforces pluripotency through a TCF-independent mechanism. Cell Stem Cell. 2011;8:214–27. 21295277 10.1016/j.stem.2010.12.010 PMC3465368

[23] Zimmerli D, Borrelli C, Jauregi-Miguel A, Söderholm S, Brütsch S, Doumpas N, et al. TBX3 acts as tissue-specific component of the Wnt/β-catenin transcriptional complex. Elife. 2020;9:e58123. 10.7554/eLife.58123 PMC7434441 32808927

[24] Mukherjee S, Luedeke DM, McCoy L, Iwafuchi M, Zorn AM. SOX transcription factors direct TCF-independent WNT/β-catenin responsive transcription to govern cell fate in human pluripotent stem cells. Cell Rep. 2022;40:111247. 36001974 10.1016/j.celrep.2022.111247 PMC10123531

[25] Fiset A, Xu E, Bergeron S, Marette A, Pelletier G, Siminovitch KA, et al. Compartmentalized CDK2 is connected with SHP-1 and β-catenin and regulates insulin internalization. Cell Signal. 2011;23:911–9. 21262353 10.1016/j.cellsig.2011.01.019

[26] Lee SH, Peng IF, Ng YG, Yanagisawa M, Bamji SX, Elia LP, et al. Synapses are regulated by the cytoplasmic tyrosine kinase Fer in a pathway mediated by p120catenin, Fer, SHP-2, and beta-catenin. J Cell Biol. 2008;183:893–908. 19047464 10.1083/jcb.200807188 PMC2592841

[27] Bahmanyar S, Kaplan DD, Deluca JG, Giddings TH Jr, O’Toole ET, Winey M, et al. beta-Catenin is a Nek2 substrate involved in centrosome separation. Genes Dev. 2008;22:91–105. 18086858 10.1101/gad.1596308 PMC2151018

[28] Dantzer C, Vaché J, Brunel A, Mahouche I, Raymond AA, Dupuy JW, et al. Emerging role of oncogenic ß-catenin in exosome biogenesis as a driver of immune escape in hepatocellular carcinoma. Elife. 2024;13:RP95191. 10.7554/eLife.95191 PMC11249736 39008536

[29] Toska E, Roberts SG. Mechanisms of transcriptional regulation by WT1 (Wilms’ tumour 1). Biochem J. 2014;461:15–32. 24927120 10.1042/BJ20131587 PMC8887836

[30] Papadopoulos D, Ha SA, Fleischhauer D, Uhl L, Russell TJ, Mikicic I, et al. The MYCN oncoprotein is an RNA-binding accessory factor of the nuclear exosome targeting complex. Mol Cell. 2024;84:2070–86.e20. 38703770 10.1016/j.molcel.2024.04.007

[31] Sato S, Idogawa M, Honda K, Fujii G, Kawashima H, Takekuma K, et al. Beta-catenin interacts with the FUS proto-oncogene product and regulates pre-mRNA splicing. Gastroenterology. 2005;129:1225–36. 16230076 10.1053/j.gastro.2005.07.025

[32] Lee HK, Choi YS, Park YA, Jeong S. Modulation of oncogenic transcription and alternative splicing by β-catenin and an RNA aptamer in colon cancer cells. Cancer Res. 2006;66:10560–6. 17079480 10.1158/0008-5472.CAN-06-2526

[33] Li Y, Chen B, Jiang X, Li Y, Wang X, Huang S, et al. A Wnt-induced lncRNA-DGCR5 splicing switch drives tumor-promoting inflammation in esophageal squamous cell carcinoma. Cell Rep. 2023;42. 10.1016/j.celrep.2023.112542 37210725

[34] Briata P, Ilengo C, Corte G, Moroni C, Rosenfeld MG, Chen CY, et al. The Wnt/beta-catenin->Pitx2 pathway controls the turnover of Pitx2 and other unstable mRNAs. Mol Cell. 2003;12:1201–11. 14636578 10.1016/s1097-2765(03)00407-6

[35] Lee HK, Jeong S. β-catenin stabilizes cyclooxygenase-2 mRNA by interacting with AU-rich elements of 3′-UTR. Nucleic Acids Res. 2006;34:5705–14. 17040897 10.1093/nar/gkl698 PMC1636482

[36] Kim I, Kwak H, Lee HK, Hyun S, Jeong S. β-Catenin recognizes a specific RNA motif in the cyclooxygenase-2 mRNA 3’-UTR and interacts with HuR in colon cancer cells. Nucleic acids Res. 2012;40:6863–72. 22544606 10.1093/nar/gks331 PMC3413138

[37] D’Uva G, Bertoni S, Lauriola M, De Carolis S, Pacilli A, D’Anello L, et al. Beta-catenin/HuR post-transcriptional machinery governs cancer stem cell features in response to hypoxia. PLoS ONE. 2013;8:e80742. 24260469 10.1371/journal.pone.0080742 PMC3829939

[38] Lee HK, Kwak HY, Hur J, Kim IA, Yang JS, Park MW, et al. β-catenin regulates multiple steps of RNA metabolism as revealed by the RNA aptamer in colon cancer cells. Cancer Res. 2007;67:9315–21. 17909039 10.1158/0008-5472.CAN-07-1128

[39] Yokoyama Y, Charnock-Jones DS, Licence D, Yanaihara A, Hastings JM, Holland CM, et al. Vascular endothelial growth factor-D is an independent prognostic factor in epithelial ovarian carcinoma. Br J Cancer. 2003;88:237–44. 12610509 10.1038/sj.bjc.6600701 PMC2377043

[40] Nakamura Y, Yasuoka H, Tsujimoto M, Yang Q, Imabun S, Nakahara M, et al. Prognostic significance of vascular endothelial growth factor D in breast carcinoma with long-term follow-up. Clin Cancer Res. 2003;9:716–21. 12576440

[41] White JD, Hewett PW, Kosuge D, McCulloch T, Enholm BC, Carmichael J, et al. Vascular endothelial growth factor-D expression is an independent prognostic marker for survival in colorectal carcinoma. Cancer Res. 2002;62:1669–75. 11912138

[42] Orlandini M, Semboloni S, Oliviero S. Beta-catenin inversely regulates vascular endothelial growth factor-D mRNA stability. J Biol Chem. 2003;278:44650–6. 12920128 10.1074/jbc.M304255200

[43] Farina AK, Bong YS, Feltes CM, Byers SW. Post-transcriptional regulation of cadherin-11 expression by GSK-3 and beta-catenin in prostate and breast cancer cells. PLoS ONE. 2009;4:e4797. 19274078 10.1371/journal.pone.0004797 PMC2650783

[44] Morgan RG, Ridsdale J, Payne M, Heesom KJ, Wilson MC, Davidson A, et al. LEF-1 drives aberrant β-catenin nuclear localization in myeloid leukemia cells. Haematologica. 2019;104:1365–77. 30630973 10.3324/haematol.2018.202846 PMC6601079

[45] Ehyai S, Miyake T, Williams D, Vinayak J, Bayfield MA, McDermott JC. FMRP recruitment of β-catenin to the translation pre-initiation complex represses translation. EMBO Rep. 2018;19:e45536. 30361391 10.15252/embr.201745536 PMC6280795

[46] Patil S, Chalkiadaki K, Mergiya TF, Krimbacher K, Amorim IS, Akerkar S, et al. eIF4E phosphorylation recruits β-catenin to mRNA cap and promotes Wnt pathway translation in dentate gyrus LTP maintenance. iScience. 2023;26:106649. 37250335 10.1016/j.isci.2023.106649 PMC10214474

[47] Balzeau J, Menezes MR, Cao S, Hagan JP The LIN28/let-7 Pathway in Cancer. Front Genet. 2017;8. 10.3389/fgene.2017.00031 PMC5368188 28400788

[48] Cai W-Y, Wei T-Z, Luo Q-C, Wu Q-W, Liu Q-F, Yang M, et al. The Wnt–β-catenin pathway represses let-7 microRNA expression through transactivation of Lin28 to augment breast cancer stem cell expansion. J Cell Sci. 2013;126:2877–89. 23613467 10.1242/jcs.123810

[49] Wagstaff M, Tsaponina O, Caalim G, Greenfield H, Milton-Harris L, Mancini EJ, et al. Crosstalk between β-catenin and WT1 signaling activity in acute myeloid leukemia. Haematologica. 2023;108:283–9. 35443562 10.3324/haematol.2021.280294 PMC9827145

[50] Wagstaff M, Sevim O, Goff A, Raynor M, Park H, Mancini EJ, et al. β-Catenin interacts with canonical RBPs including MSI2 to associate with a Wnt signalling mRNA network in myeloid leukaemia cells. Oncogene. 2025. 10.1038/s41388-025-03415-y. 10.1038/s41388-025-03415-y PMC12256266 40301545

[51] Panelli P, De Santis E, Colucci M, Tamiro F, Sansico F, Miroballo M, et al. Noncanonical β-catenin interactions promote leukemia-initiating activity in early T-cell acute lymphoblastic leukemia. Blood. 2023;141:1597–609. 36315912 10.1182/blood.2022017079 PMC10651788

[52] Wagstaff M, Coke B, Hodgkiss GR, Morgan RG Targeting β-catenin in acute myeloid leukaemia: past, present, and future perspectives. Biosci Rep. 2022;42. 10.1042/BSR20211841 PMC9069440 35352805

[53] Gebauer F, Schwarzl T, Valcárcel J, Hentze MW. RNA-binding proteins in human genetic disease. Nat Rev Genet. 2021;22:185–98. 33235359 10.1038/s41576-020-00302-y

[54] Kang D, Lee Y, Lee JS. RNA-binding proteins in cancer: functional and therapeutic perspectives. Cancers. 2020;12. 10.3390/cancers12092699 PMC7563379 32967226

[55] Edwards TA, Pyle SE, Wharton RP, Aggarwal AK. Structure of Pumilio reveals similarity between RNA and peptide binding motifs. Cell. 2001;105:281–9. 11336677 10.1016/s0092-8674(01)00318-x

[56] Moore S, Jarvelin AI, Davis I, Bond GL, Castello A. Expanding horizons: new roles for non-canonical RNA-binding proteins in cancer. Curr Opin Genet Dev. 2018;48:112–20. 29216518 10.1016/j.gde.2017.11.006 PMC5894799

[57] Yang Z, Zhou J, Li Z, Guo J, Fang L, Xiao X, et al. Identification of whole-cell dsRNA-binding proteins by phase separation. RNA Biol. 2024;21:32–45. 39115224 10.1080/15476286.2024.2386498 PMC11312991

[58] Castello A, Fischer B, Eichelbaum K, Horos R, Beckmann BM, Strein C, et al. Insights into RNA biology from an atlas of mammalian mRNA-binding proteins. Cell. 2012;149:1393–406. 22658674 10.1016/j.cell.2012.04.031

[59] Ule J, Jensen KB, Ruggiu M, Mele A, Ule A, Darnell RB. CLIP identifies Nova-regulated RNA networks in the brain. Science. 2003;302:1212–5. 14615540 10.1126/science.1090095

[60] García-Hernández V, Arambilet D, Guillén Y, Lobo-Jarne T, Maqueda M, Gekas C, et al. β-Catenin activity induces an RNA biosynthesis program promoting therapy resistance in T-cell acute lymphoblastic leukemia. EMBO Mol Med. 2023;15:e16554. 36597789 10.15252/emmm.202216554 PMC9906382

[61] Gui T, Fleming C, Manzato C, Bourgeois B, Sirati N, Heuer J, et al. Targeted perturbation of signaling-driven condensates. Mol Cell. 2023;83:4141–57.e11. 37977121 10.1016/j.molcel.2023.10.023

[62] Storci G, Bertoni S, De Carolis S, Papi A, Nati M, Ceccarelli C. et al. Slug/β-catenin-dependentproinflammatory phenotype in hypoxic breast cancer stem cells. Am J Pathol. 2013;183:1688–97. 24036252 10.1016/j.ajpath.2013.07.020

